# High-Definition Survey of Architectural Heritage Fusing Multisensors—The Case of Beamless Hall at Linggu Temple in Nanjing, China

**DOI:** 10.3390/s22093369

**Published:** 2022-04-28

**Authors:** Junwei Fang, Yingying Zhang, Yiru Zhang, Huayu Guo, Zheng Sun

**Affiliations:** School of Architecture, Nanjing Tech University, Nanjing 211800, China; 202061211026@njtech.edu.cn (J.F.); zhangyingying@njtech.edu.cn (Y.Z.); 202061211044@njtech.edu.cn (Y.Z.); ghy812@njtech.edu.cn (H.G.)

**Keywords:** architectural heritage survey, damage mapping, laser scanning, photogrammetry, brick masonry, architectural graphics

## Abstract

Following the development of digital measurement technology in recent years, the information contained in the measurement outcomes have become increasingly rich. However, the traditional graphical representation method based on vector graph needs to be updated. In this study, we use the Beamless Hall of Linggu Temple as an example. Measurements are conducted by using digital techniques, including three-dimensional (3D) laser scanning, close-range photogrammetry, and infrared thermal imaging. The pseudocolours that express spatial information and moisture distribution are calculated and generated through point clouds, which are used to express the land subsidence, wall deformation, moisture distribution, and other effects of the Beamless Hall. Furthermore, combining it with two-dimensional (2D) graphical representation, such as the plan, elevation, and section, damage-related information can be expressed intuitively and efficiently. This method can combine the advantages of graphics and images to provide a comprehensive and intuitive representation of the digital measurement results of brick architecture heritage. It can also provide a reference for surveying similar monuments and buildings of our architectural heritage.

## 1. Introduction

Architectural heritage surveying and mapping is a significant foundation and prerequisite for architectural heritage conservation and research on architectural history, which refers to the application of the principles and methods of surveying and graphics to collect and express the spatial geometric information of architectural heritage [1]. In the process of surveying and mapping, heritage conservation involves various types of information in addition to the ontological information of the building. Following the development of digital technologies in recent years, such as satellite remote sensing, global positioning system, and three-dimensional (3D) laser scanning and photogrammetry, the accuracy, efficiency, and integrity of architectural heritage surveying and mapping have improved considerably. At the same time, the application of these digital technologies also enriches considerably the expression form of the measurement outcomes. In addition, comprehensive and multidisciplinary characteristics have been presented. Traditional surveying and mapping drawings based on two-dimensional (2D) vectors, such as plans, elevations, and sections, can express geometry, texture, damages, and other information, but there are problems, such as data dimensionality reduction and data dispersion. The raw result of 3D laser scanning, photogrammetry, and other digital technologies include digital elevation models, 3D point-cloud models, scanned reflection intensity data, infrared thermal images, and others. The traditional field of architectural heritage surveying and mapping is suffering from the impact of digital technology’s development owing to the increasing abundance of measurements achieved by digital technology. This has divided the process of surveying and mapping into two separate processes: the process of surveying, often being handled by specialised technicians, and the mapping drawings remaining the responsibility of architects. The problem, however, pertains to the fact that technicians do not often understand the architectural needs. This leads to the use of measured data, which are not exploited to their full potential, while people with architectural backgrounds are confused about the benefits of digital technology. Exploring new methods of graphical representation is necessary for the expression of the results of architectural heritage surveying and mapping.

In China, architectural heritage surveying and mapping with modern measurement devices and engineering graphics originated in the 1930s. Currently, surveying and mapping is classified into comprehensive, typical, and abbreviated surveying and mapping according to the level of surveying and mapping [2]. The three categories are executed according to different purposes (i.e., research on architectural history, restoration, and protection). The results of the three categories of surveying records are mainly 2D graphs and images, with the images currently being mainly in the form of photographs, which serve as records. Owing to the development of computer vision, algorithms, and other technologies in recent years, the results of advanced measurement tools can be expressed through images processed by computer technology. Currently, the image obtained as a result of surveying and mapping has a high accuracy. More importantly, such an image can provide support for the qualitative to the quantitative analysis in the surveying and mapping of architectural heritage.

The types of destructions suffered by the brick masonry studied herein are mainly structural destructions and detrimental effects of the masonry structure materials themselves [3]. At present, many studies have been published on the pathology of brick masonry [4,5], whereas only few studies have been reported on the graphical representation of brick masonry damages. In previous typical surveying and mapping results, it was difficult to visualise the damage conditions of most brick masonries because important types of architectural heritage suffer from various detrimental effects. Therefore, it is urgent to explore a graphical representation method of the destruction of brick architectural heritage. The research in this study is based on the specific needs in architectural heritage surveying and mapping. Ground subsidence, wall deformation, and moisture distribution are analysed using point-cloud data, after which the graphical representation of the brick architecture heritage is explored in the form of results, which combine graphs and images.

### Research Aim

The graphical representation method used for recording the as-built conditions of the Chinese historic architecture is still in its infancy. The aim of the present study is to develop a CAD-based workflow leveraging the power of digital surveying in historic architecture conservation projects in China. Specifically, it investigates ways to combine digital survey results collected with terrestrial laser scanning (TLS), photogrammetry, and infrared thermal camera with traditional mapping of plans, elevations, and section drawings and with 3D CAD models for representation. Such a workflow is fast, intuitive, and friendly to nonexpert users in BIM. This way, the CAD-based vector drawings integrated with pseudocolour images could represent the structural deviation, moisture content, and other building pathologies. We hope that the case studied herein (i.e., the Beamless Hall at Linggu Temple) could serve as a universal approach to the graphical representation of brick heritage surveying and mapping and provide an effective reference and aid for practical restoration and conservation projects.

## 2. Related Works

### 2.1. Point-Cloud Processing

TLS has been used to acquire high-resolution spatial information of the built heritage since 1990s as an efficient and noncontact method. The output of TLS is known as the point cloud. A vast literature exists on extracting conservation-relevant information (e.g., structural deformation and surface pathology) based on point-cloud computation.

Pepe et al. [6] generated point-cloud models of measured objects (a church with brick and stone bridges) by photogrammetry and 3D laser scanning techniques. They then built a current 3D model with the use of the software Rhinoceros (version 6, manufacturer: Robert McNeel & Assoc, Seattle, Washington, DC, USA). Based on this model, the structures of the church and the stone bridge were studied and discussed using finite element analyses. Banfi et al. [7] discussed the automation of the modelling process from the point-cloud model to the BIM model and performed finite element analyses of the studied object based on the BIM model. Pesci et al. [8] studied the palace of Accursio by means of red–green–blue (RGB) information and geometric information obtained by 3D laser scanning and high-resolution digital imaging techniques, with the aim of inferring original architectural information about the ancient site of the palace.

Costa-Jover et al. [9] discussed and analysed the structure of the vaults by means of 2D and 3D assessments based on the data acquisition of the Tortosa Cathedral using a 3D laser scanner. The 3D evaluation was conducted by modelling the church’s construction information and records over time and then comparing the vault heights for each period. Two-dimensional evaluation was performed by plotting the values reflected in the height histograms of each vault and by drawing the horizontal and vertical profile lines for comparison.

Most TLS systems also record intensity data, loosely defined as the strength of the backscattered echo for each measured point [10]. In the field of built heritage, TLS-intensity data are promising as a noninvasive approach in detecting and monitoring surface damages [11,12]. The intensity of TLS is relevant to data acquisition geometry, environmental effects, target surface characteristics, and instrumental effects [10]. Theoretically the intensity could be determined by the radar equation (Equation (1)).

(1) $$P_{r}=\frac{P_{t\;}D_{r}{}^{2}\eta_{\mathrm{atm}}\eta_{\mathrm{sys}}\sigma }{4{\pi R}^{4}\beta_{t}{}^{2}}$$

where P_r_ = received optical power, P_t_ = transmitted power, D_r_ = receiver aperture diameter, σ = effective target cross section, η_atm_ = atmospheric transmission factor, η_sys_ = system transmission factor, R = range, and β_t_ = transmit beamwidth.

In practice, the instrumental data can be considered as constants (i.e., P_t_, D_r_, and η_sys_) when using the same laser scanner, and the atmospheric transmission effect can be neglected for close-range TLS. Accordingly, Equation (1) can be simplified to the form of Equation (2) [13]:

(2) $$P_{r}=C\;\times \;\rho \;\times \;\cos\theta \;\times R^{-2}$$

where C = P_t_ D_r_ ηsys/4 is an unknown but constant parameter for a specific scanner, ρ = the reflectance of the object surface, and θ = the incidence angle.

According to the Equation (2), the intensity of TLS is proportional to the cosine of the incidence angle and inversely proportional to the range squared. Many studies conclude that the range and incidence angle are independent of each other and can be corrected separately [14,15]. Existing correction methods can be categorised as model-driven and data-driven [16]. A global correction is still difficult owing to various issues, such as surface roughness, but a local correction within certain scanning distance and incidence angle is possible. This gives rise to a discrimination of brick surface characteristics (e.g., dating, weathering degree, moisture, and salt) at the millimetre level.

### 2.2. Representation with Computer Graphics

From the perspective of theoretical studies of graphical representation, Wu et al. [17] described the specific concept of architectural graphics and the history of architectural drafting, based on which they provided a detailed analysis of the current dilemma of architectural graphics. Li [18] reviewed and analysed the representation of traditional architectural heritage surveying and mapping results and current surveying and mapping results. On this basis, Li proposed the method of visualisation, interactive expression, and information management of architectural heritage information by using GIS and BIM as the core technologies that realised the management of building ontology information through BIM while using GIS as a management tool of spatial environment information.

In the practice of architectural heritage conservation, CAD is used more in the pre-processing stage of surveying data and as a basis for data integration or further analysis in other platforms [19]. Giunta et al. [20] conducted measurements on the Milan Cathedral by terrestrial laser scanning, photogrammetry, thermographic, and geo-radar, based on which a 3D CAD model was created. Vector drawings with different themes were then created according to the different types of measurements. On this basis, all the data were integrated in a geo-referenced and integrated 3D database of the Cathedral. Giammartini et al. [21] measured the building facades of the medieval St. Giuliana and used CAD for the visual representation. This was mainly used for recording the rock types and for image processing and storage based on vector drawings. Lezzerini et al. [22] used St. Nicholas as a practical case. In their study, digital mapping was first conducted by means of a 3D laser scanner followed by a study of the stone of the church’s facade based on the use of GIS, CAD, and high-resolution images. In addition, the study highlighted the potential of using CAD and GIS in this architectural practice.

From the perspective of GIS applications in architectural heritage conservation, Campanaro et al. [23] proposed the “white box” workflow by combining digital measurement technology with 3D GIS. Effectively, these authors combined 2D measured, data processing outcomes (including stress analysis, building damage, and geometric information) with 3D models and performed query and management functions. He et al. [24] proposed a GIS-based approach to document and analyse cultural heritage at continuous spatial scales. For historic buildings, courtyards, historic towns, and archaeological sites, they developed a systematic approach for specific conservation studies in terms of historical information systems. Currently, GIS is more often used in heritage conservation practices at the macro level [25,26,27]. In addition, GIS is becoming one of the most influential tools in the field of architectural heritage mapping owing to the advantage of being able to manage, connect, and analyse different types of data information [28,29,30,31].

In terms of BIM, Simeone et al. [32] developed a BIM semantic-enrichment approach that integrated BIM with a knowledge database developed through information ontologies to meet the semantic requirements for building heritage information models. Bruno et al. [33] implemented a systematic BIM process for the case of Parma Cathedral, which included the modelling of irregular shapes, historical data management, engineering management, and platform query functions, with the aim of developing a flexible and targeted approach to architectural heritage planning to apply it to architectural heritage conservation practices. Godinho et al. [34] presented the development of BIM and used the example of the National Palace of Sintra in Portugal to create a complete database and 3D model through BIM, in addition to the proposed two-way information flow design of the system, which supported data acquisition, modelling, retrieval, and updating. Moreover, the model was also able to interact with structural analysis software based on real records. BIM has not been originally developed as a software for use in architectural heritage, but it has assumed an increasing role in the conservation of architectural heritage [35].

Furthermore, López et al. [36] discussed the limitations and possibilities of applying BIM platforms in architectural heritage restoration projects based on a comprehensive review of 131 papers on maintenance, management, and parametric BIM. The article concluded that using BIM in conjunction with GIS and other supporting software is an effective solution for managing and modelling point-cloud data and semantic information in a semiautomatic manner. Logothetis et al. [37] enriched the digital expression of BIM by developing an open-source platform that supported historic building information modelling (HBIM). The authors developed a plug-in for BIM within the Free and Open-Source Software (FOSS) CAD environment, which enabled the import and editing of surveying results obtained through photogrammetry techniques in BIM.

The use of a single platform often has many limitations, and it is desirable to combine the advantages of multiple platforms used together according to actual needs.

### 2.3. Level of Detail

The scale of representation is one of the core issues in digital recording and analysis of architectural heritage. In a manual survey, one should always bear in mind the scale of drawing delivery when conducting measurements. For a CAD-based representation, it is wise to consider the smallest plottable detail (0.2 mm) [38]. The uses of TLS and photogrammetry, however, often capture highly dense data in the form of 3D point clouds and 2D images. Some studies have been devoted to the scan-to-BIM process and its level of detail (LoD) in the past decade [39,40]. In the geometric translation process from point clouds to BIM objects, the loss of geometric accuracy is difficult to avoid, and high labour intensity is required.

In some survey and restoration projects, especially at their early stages when only 2D drawings are demanded, a CAD-based 3D model integrated with the point cloud could speed up the workflow. Instead of as-built 3D modelling in BIM, the geometric deformation and other damages could be represented by taking advantage of dense point clouds and their processing algorithms.

### 2.4. Summary

An issue that has created widespread concern in the field of architectural heritage is the efficient processing and representation of the results of digital measurements. Geometric accuracy, resolution (LoD), and automation are highly valued during this process. Many research efforts have been expended to intelligently extract conservation-relevant knowledge from point-cloud data. These efforts have created opportunities for elaborate data management, retrieval, and sharing. Meanwhile, extensive research efforts have focused on the application of BIM and GIS, and the standards for BIM and GIS are well mature in European countries such as Italy. Nonetheless, the use of CAD is still dominant in architectural heritage surveying and mapping in China. This is not only because of the approval requirements for restoration and conservation projects in China but also because most practitioners are currently accustomed to the use of CAD. Therefore, the research in this paper focuses on the combination of digital measurement tools and CAD representation, which is of some practical significance. Point-cloud processing integrated with 2D drawings is expected to speed up the survey workflow and enhance its clarity to stakeholders, including nonexpert users.

## 3. Data Collection

### 3.1. Description of the Beamless Hall at Linggu Temple

**Figure 1:**
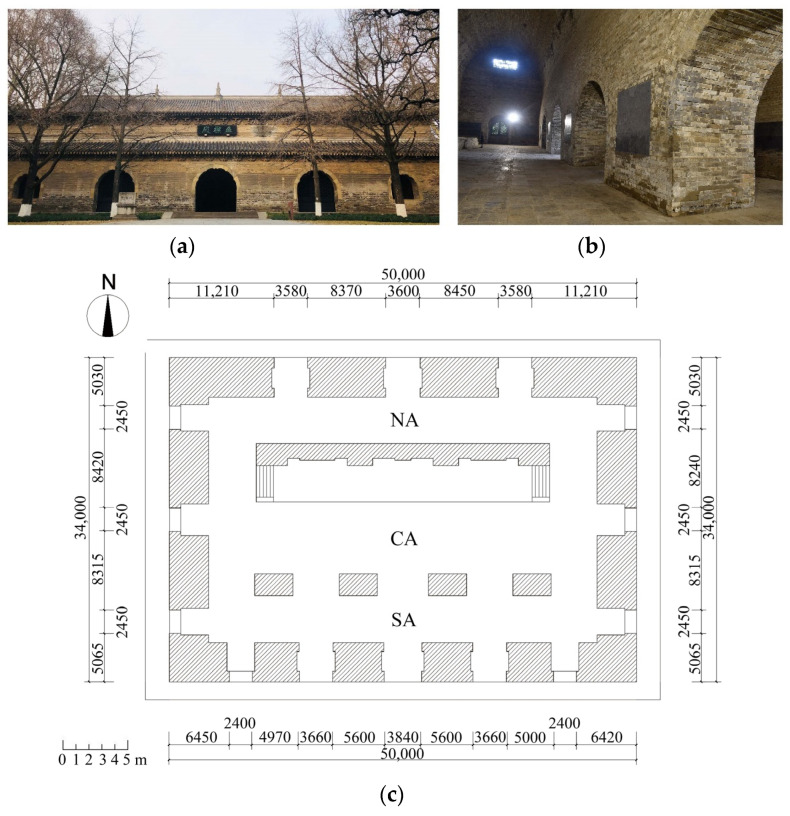
Beamless Hall at Linggu Temple: (**a**) exterior view, (**b**) interior view, and (**c**) floor plan.

The building studied in the present work (the Beamless Hall at Linggu Temple) is located in Nanjing in China (Figure 1). Beamless Hall is believed to have been built as a Buddhist temple in the 14th or 15th centuries. The volume of the building was pierced by a series of barrel vaults and arches made of bricks and mortar. Along the longitudinal axis, there are three parallel spaces with lengths equal to 40 m spanned by three-barrel vaults. The dominating central vault (CA) has a span > 11 m, one of the largest among similar brick-masonry vaulted structures built in the 14th to 15th centuries in China. Standing on the south and north of the CA are two smaller barrel vaults (south vault: SA and the north vault: NA). As a result of natural influences and human activities over the last hundred years, the Beamless Hall of Linggu Temple is suffering from material weathering, structural deformation cracks, and moisture [41].

**Figure 2:**
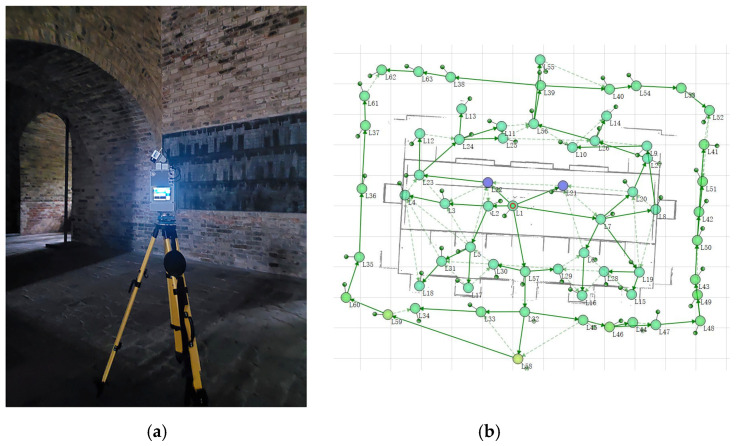
Terrestrial laser-scanner-based survey for the Beamless Hall. (**a**) Field survey with a Z + F Imager 5010X laser scanner. (**b**) Spatial distribution of 63 scan stations.

In October 2020, we obtained the 3D geometric, 2D image, and thermal infrared information of the Beamless Hall by applying 3D laser scanning technology (Figure 2) and photogrammetry technology. On this basis, we discussed the deterioration of the Beamless Hall and conducted a study on the graphical representation method of the brick masonry.

### 3.2. Measurement Techniques Used

#### 3.2.1. 3D Laser Scanning

In recent years, 3D laser scanning technology has been increasingly used in architectural heritage conservation [42]. The device of terrestrial laser scanning used in this study is Z + F 5010X, and the scanner is also equipped with a thermal infrared camera (model: Z + F T-Cam) to collect infrared thermal image information of the Beamless Hall of Linggu Temple.

In point clouds acquired by the terrestrial laser scanner, individual point-cloud models can contain geometric, colour, intensity, and temperature information (Equation (3)).

P_n_ = {X_n_, Y_n_, Z_n_, R_n_, G_n_, B_n_, I_n_, T_n_}, (3)

where P_n_ is the set of point-cloud models with number n. X_n_, Y_n_, and Z_n_ are the X, Y, and Z coordinates of the point cloud, respectively, R_n_, G_n_, and B_n_ are the colours of the point cloud, I_n_ is the intensity value of the point cloud, and T_n_ is the temperature value of the point cloud. In addition to its capacity to serve as references for drawing and modelling, the inherent features of the point cloud are computed and utilised in this study.

#### 3.2.2. Photogrammetry

Photogrammetry is one of the 3D modelling methods used in the field of architectural heritage surveying and mapping and is an image-based 3D reconstruction technique [43]. In the practical application of the Beamless Hall at Linggu Temple, we used a camera (model: Nikon D5600) to photograph the interior of the Beamless Hall and then created a 3D point-cloud model in the software Metashape, (version:1.6.4, manufacturer: Robert McNeel & Assoc, St. Petersburg, Russia) following the steps listed below:

- Align photos: default (accuracy: medium; key point limit: 40,000, tie point limit: 4000).
- Build dense cloud: default (quality: medium).
- Build mesh: default (quality: medium; face count: medium).
- Build texture: default (texture type: diffuse map; mapping mode: generic).
- Build orthomosaic: default (type: planar; surface: mesh).

The point-cloud/mesh model was able to record the texture and colour of the interior walls of the Beamless Hall. This accounted for the deficiencies of the terrestrial laser scanner in this aspect and laid the foundation for exploring the visual representation of the deterioration.

#### 3.2.3. Infrared Thermal Image

In brick masonry, the determination of moisture deterioration is critical for making repair decisions [44]. At present, infrared thermal imaging is extensively used in the detection of building pathologies [45,46], but the expression form of the results remains to be explored.

Therefore, based on the infrared thermal image information collected by the terrestrial laser scanner, this study discusses its application in architectural heritage conservation and the graphics representation method accordingly.

In the morning of 26 October 2020, we collected infrared thermal image information from the Beamless Hall using a thermal infrared camera (Z + F T-Cam, Table 1) equipped on a terrestrial laser scanner. Furthermore, the weather condition on that day was cloudy and the temperature was in the range of 12–21 °C.

## 4. Graphical Representation of Architectural Pathologies

**Figure 3:**
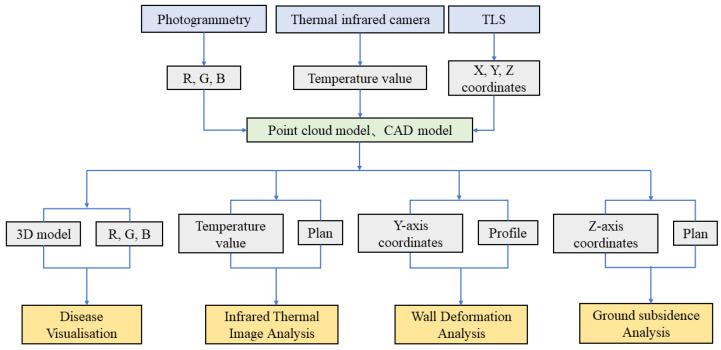
Pipeline of the proposed method.

The graphical representation used herein is a combination of 2D vector lines drawn in CAD programmes and the results of images processed through computer technology (Table 2). Among them, the plan and section of the Beamless Hall are drawn in AutoCAD with reference to the relevant data of the point-cloud model (Figure 3).

### 4.1. Ground Subsidence Analysis

In the drawing process of the plan, the plane contour obtained by intercepting the point-cloud model has the characteristics of high accuracy compared with the plane contour obtained by using traditional measuring tools, such as tape measures and laser rangefinders.

**Figure 4:**
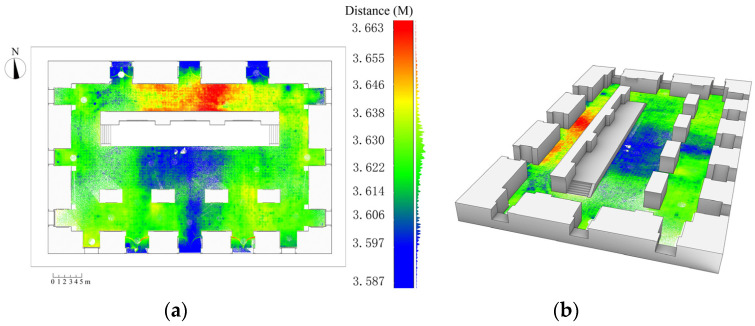
Ground subsidence analysis of the beamless hall of Linggu Temple. (**a**) Two-dimensional (2D) and (**b**) three-dimensional (3D) expression of subsidence analysis.

In addition, the representation of ground subsidence is significant in the expression of the plan. By converting the Z-coordinates (height) of the point-cloud model into a pseudocolour scale, an image showing information on the uneven ground subsidence of the Beamless Hall can be obtained and then superimposed on the plan. Moreover, by combining with the images of the 3D model, it can reflect the subsidence information more intuitively. From Figure 4, it can be observed that:

(a) The most obvious uneven subsidence of the ground of the Beamless Hall is in the middle ground of the north arch (NA) and the section of the ground from the south arch (SA) into the central arch (CA); the difference between the two subsidence distances is approximately 8 cm.
(b) There is an obvious ground subsidence at the three entrances of the SA and NA, and the value of ground subsidence at the entrance is approximately 5 cm compared with the average height of the interior floor of the Beamless Hall.

### 4.2. Wall Deformation Analysis

The point-cloud model generated by 3D laser scanning and photogrammetry can satisfy the needs of studying the deformation of building structures. Like drawing plan contours, building sections drawn by intercepting contours of point-cloud models also have high accuracy. In addition, in brick masonry, the extent and degree of wall deformation is what needs to be highlighted in the section, which often requires a higher degree of accuracy and density. However, traditional architectural heritage surveying and mapping is hardly capable of handling these needs. Based on the point-cloud model obtained by digital measurement technology, the wall deformation can be studied by cutting the 3D point-cloud model of the measured building, calculating geometric features and other operations. Additionally, according to the higher standard of surveying and mapping requirements, the scatter diagram can be drawn for the parts with serious structural deformation, and the accuracy can reach the centimetre level, which can reflect the deformation information more accurately.

**Figure 5:**
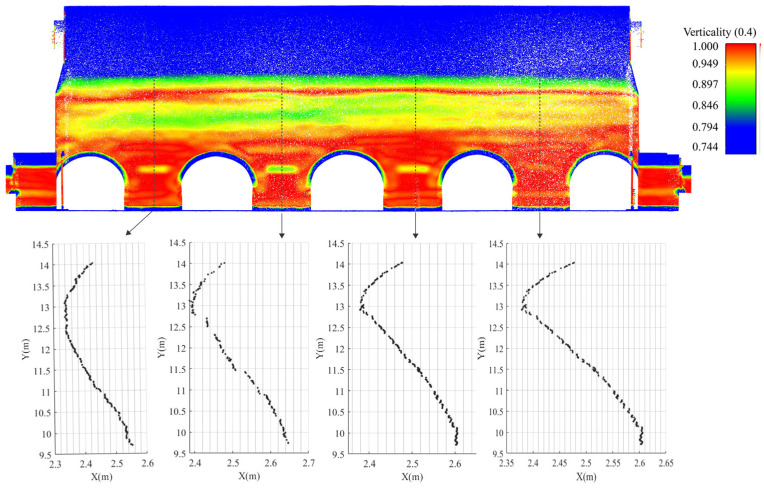
South wall deformation analysis of CA. The top of the image shows the verticality analysis of the wall surface, and the bottom shows the scatter diagram of the local section of the wall.

In addition, the corresponding information can be reflected by studying the geometric features of the point cloud. The pseudocolours in Figure 5 come from the calculation of the verticality (Equation (4)) of the wall, which is achieved by using the open-source software CloudCompare (Version 2.12 beta (windows 64-bit). 2021. Retrieved from http://www.cloudcompare.org/, accessed on 26 July 2021) to calculate the geometric features of the point cloud that has a radius set to 0.4 m. The geometric features (i.e., flatness, perpendicularity, and sphericity) of the point cloud are described as a combination of eigenvalues (λ1 > λ2 > λ3) [47] extracted from the covariance matrix. In this study, the wall verticality is calculated to represent the wall deformation of the SA in the Beamless Hall.

V = 1 − n_Z_ (4)

where V = verticality and n_Z_ = the “z” component of the normal vector “n”.

**Figure 6:**
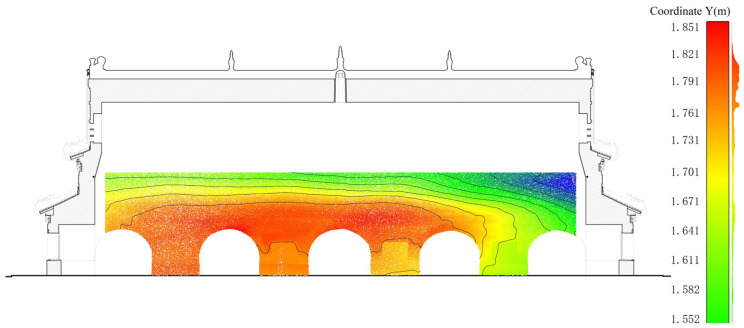
Horizontal displacement analysis of the south wall of CA (pseudocolour: offset value in the Y-axis direction; contour: contour spacing of 0.05 m).

Finally, we tried to combine the 2D sections with the images to have a visual expression of the deterioration information. The pseudocolour in Figure 6 expresses the Y-axis offset value of the south wall of the CA, and the vector lines in Figure 6 are contour lines with a spacing of 0.05 m.

Through this series of sections, we learned that:

(a) The verticality calculation of the six walls showed that the south wall suffered more extensive deformations than the north wall;
(b) An S-shape distortion was observed at the south wall under CA (Figure 5). It is probably owing to the interacted thrust between SA and CA. The former thrust led to a lateral displacement (ca. 10 cm) around the springing of SA, and the latter led to a reverse displacement (ca. 15 cm) around the springing of CA.

### 4.3. Infrared Thermal Image Analysis

In the field of architecture, infrared thermal imaging mainly detects thermal defects, exterior wall voids, cracks, and moisture destructions in buildings [48]. Among them, moisture deterioration is the main type of damage suffered by brick masonry, which is especially evident on the top of the north arch of the Beamless Hall, but the moisture areas are difficult to locate by conventional means. However, when the building materials are moistened, their heat capacities and thermal conductivities increase. At this time, when the outdoor environment (solar radiation and air temperature) changes, there is a significant temperature difference between the moistened parts and the surrounding dry parts, and this temperature difference is reflected in the infrared thermogram as a difference in colours [49]. Therefore, based on this method, the moisture area can be located accurately and then combined with the surrounding environment and building structural information. Accordingly, the damage type can be speculated, and the corresponding protection plan can be formulated.

At the same time, the application of infrared thermal image also provides support for this deterioration type from one type of qualitative analysis to another. During the fieldwork of the Beamless Hall of Linggu Temple, the rainwater leakage and moisture effects in the north arch vault were the most obvious.

**Figure 7:**
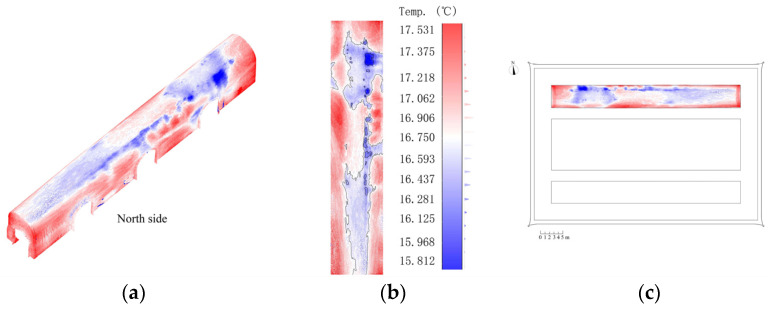
Moisture distribution analysis at the top of NA (pseudocolours show the temperature values). (**a**) Three-dimensional point-cloud model. (**b**) View of vault expansion. (**c**) Bottom view.

In this study, the distribution of moisture areas is analysed based on the infrared thermal image information contained in the point-cloud model of the NA. First, a combination of images and graphs was used to combine the indoor bottom view of the Beamless Hall with images containing infrared thermal image information. Moreover, the vault of the NA was expanded to convert 3D point-cloud model image into a 2D point-cloud model image. On this basis, the moisture distribution area is plotted by using vector lines so that a more intuitive expression of the moisture part of the NA can be made. From Figure 7, it can be observed that:

- The overall temperature range of the NA was between 15.5 °C and 18 °C.
- Moisture was prevalent in the top of the arch of the NA with the most severe part of the arch located on the north side of the west arch top of the NA.

### 4.4. Visualisation of Deterioration

The conservation of architectural heritage requires the participation of professional people but also the involvement of the entire society. The traditional method of graphical representation of architectural heritage does not satisfy the need to express the increasingly abundant measurement data so that information about the significance, value, and deterioration suffered by architectural heritage cannot be conveyed visually. However, the development of digital technology has brought new opportunities for innovation in the graphical representation of this field.

In this study, the deformation information of the wall and the texture mesh from photogrammetry were combined with a 3D model to create a sectional perspective view. This was performed not only to explore an enhanced 3D visual representation of the damage but also to be able to communicate the deterioration information of the building’s body in a general way and to provide a direct help for professionals to execute their work.

**Figure 8:**
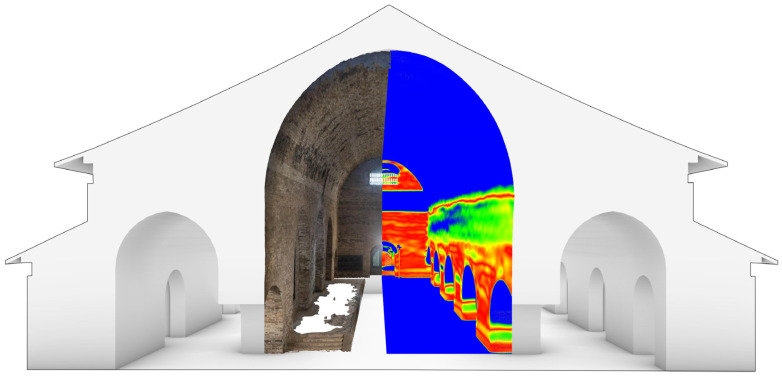
Visual expression of the deformation area of the south wall of CA (the true colour of CA in the figure is from photogrammetry, and the pseudocolour indicates the verticality of the wall).

By combining the verticality calculation and the texture mesh obtained through photogrammetry, it is possible to visualise the area and extent of wall deformation (Figure 8). The expression form of deterioration visualisation is not limited to this, but it is important to illustrate the deterioration with vivid images. In practical applications, specific expressions can be made according to the disease condition of specific building types.

## 5. Discussion

Architectural heritage surveying and mapping is an important foundation for architectural heritage conservation and research on architectural history. Graphical representation in architectural heritage surveying and mapping fundamentally aims at conveying information about architectural heritage to conservation stakeholders (architects, engineers, architectural historians, restorers, etc.). With the emergence of new measurement technologies, the form of graphical representation in architectural heritage surveying and mapping has changed, mainly owing to the increase in the number of information carriers, types of information, and the amount of information. TLS, infrared thermography, and photogrammetry collect as-built information of a building with high precision and resolution, but 2D vectors are invalid to graphically represent such information comprehensively. Although BIM has attracted much research attention in the field of built heritage owing to associative drawings, data management, and encouragement to structural simulations, using BIM in a restoration project one has to consider a series of issues such as essential skills, establishing the component library, and labour intensity. Currently, 2D vector drawings using CAD are still the dominant representation method, especially in the preliminary stage of a restoration project in China.

Another consideration of the proposed study is how to effectively represent the technical assessment. The technical assessment prior to the start of architectural heritage restoration and conservation works (which includes building moisture detection, foundation detection, and structural deformation detection) is mainly carried out by professional technicians. The lateral displacement of the building’s structure is often measured with a total station from a geodetic network. In spite of its accuracy and low cost, the results of this method highly depend on the operator’s skill and the choice of observed sampling points. Our method proposes a straightforward approach for detecting and representing structural displacement based on point-cloud computation. The method could speed up the technical assessment of architectural heritage, especially for those structures that have been scanned by TLS, and clouds improve the analysis of structural displacement globally. Similarly, our method facilitates the nondestructive detection of moisture content and rainwater leakage, which are usually (nowadays) detected by infrared thermal imaging that requires further processing for metric data retrieval [50]. Furthermore, due to their different disciplinary backgrounds, their graphical representation does not allow for an intuitive and efficient representation of building information. Instead of serving as a measurement approach for drawings, the integrated uses of TLS, infrared thermography, and photogrammetry could give rise to the detection of structural deformation and moisture content.

**Figure 9:**
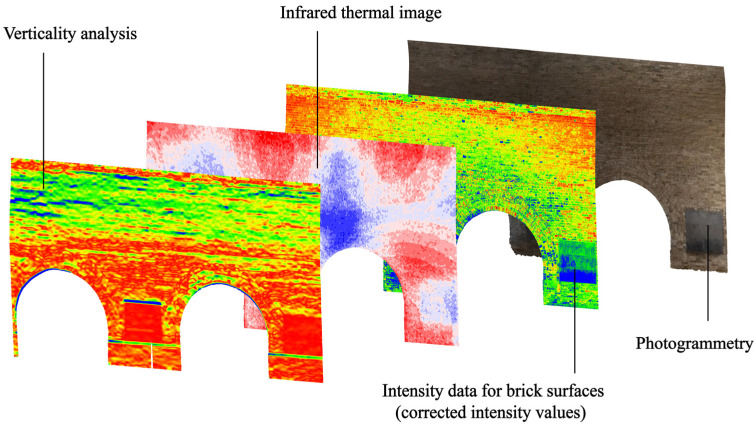
Expression of results using digital measurement technology.

Our study proposed a workflow of graphical representing multisensors collected data for architectural heritage conservation. The graphical representation method described in this paper mainly combines images from 2D vector drawings and digital measurement results, which can meet the needs of both macroscopic and microscopic levels of expression, as well as interpreting architectural heritage from an architectural perspective as a means of conveying architectural information. At the same time, by combining the results of different measurement tools, it is possible to carry out comprehensive studies on building pathologies and structures (Figure 9), which to a certain extent provides a more effective aid to architectural heritage restoration and conservation projects. Although the established 3D model in this study is featured with limited architectural details to speed up the workflow, it provides a flexible framework to which the optically measured image data could be registered and visualised at multiresolutions. For example, the infrared thermal images have approximate m-level resolution owing to the camera’s pixel and scanning distance, while the intensity image’s resolution is at the millimetre level, thus allowing the execution of additional, elaborate studies. The integrated use of both data (3D CAD model and 2D images) allows intuitive damage visualisation with lightweight files, which is favourable for web-based applications.

The ground subsidence analysis allows the observation of the information on uneven ground subsidence. The deformation analysis of walls can meet the needs of studying the deformation of building structures. The integration of these two aspects provides clues for elaborated structural analysis coupled with historic restoration archives, finite element analysis, and other nondestructive testing, such as ground penetrating radar. Infrared thermal image analysis can provide the basis for quantitative analysis of moisture damage treatment of brick building heritage. The visual expression of the destruction is also intended to be able to convey more intuitive information about the destruction of the building. In addition, it is important to note that the measurement technology is always advancing, and the graphical representation of architecture should be improved.

This study has some limitations. This study analyses and discusses the ground subsidence, wall deformation, and moisture distribution of brick masonry. The research in this study mainly focused on the analysis based on the information collected from the building’s surface. Owing to the structural characteristics of brick masonry, a more accurate inference needs to be made in combination with the internal structure. Therefore, in the future, we will consider the use of ultrasonic and microwave technologies to collect data from the interior parts of the building and then combine the information on the building surface for a comprehensive analysis. In terms of graphical representation, future research will be based on point-cloud models to further improve the accuracy of the CAD results. In addition, as the object of study is a masonry building, the contours of the bricks will also be automatically extracted in the point-cloud model by means of an algorithm, based on which quantitative statistics will be combined with the images. Additional structural analysis will be carried out by drawing cross-sections of the wall construction.

## 6. Conclusions

New methods of graphical representation of surveying and mapping results make the content of drawings for architectural heritage surveying and mapping more intuitive, and they contain more information than ever before. Surveying technology is always advancing, and the graphical representation of architecture has to continue to improve.

This article discusses the graphical innovation of architectural heritage surveying and mapping with the example of the brick masonry of the Beamless Hall in Linggu Temple. The main conclusions of this study are as follows:

(a) Architectural heritage surveying and mapping is an important approach that allows intervention in heritage conservation. The advantage of architecture involves the interpretation of the surveyed object from the perspective of architectural discipline, while the replacement of measuring tools should not be a factor that would affect the dominant position of architectures in this field.
(b) The ground subsidence diagram, wall deformation analysis diagram, and moisture distribution analysis diagram can reflect visually the situation and extent of destruction that the building is suffering from and provide guidance for the subsequent conservation decision stage.
(c) The subsequent step is to establish the current situation model based on the point-cloud model, after which the information of graphics and images are integrated with the 3D model in the BIM platform aiming at creating a systematic and intuitive data management strategy. On this basis, visual interaction is provided for data users (heritage manager, experts, and visitors).

## Figures and Tables

**Table 1:** Technical data of the thermal infrared camera used in this paper.

Parameter	Value
Resolution	382 × 288 pixels
Infrared spectrum	7.5–13 μm
Working temperature	0–50 °C (32–122 °F)
Storage temperature	−20–60 °C (−4–140 °F)
Working range	>1.6 m (4.8 ft)
Temperature resolution	4096 increments (12 Bit)
System accuracy/absolute temperature accuracy	1 ± 2 °C

**Table 2:** Summary table of the point-cloud information used in the graphics representation of this study.

Serial Number	Name	Point-Cloud Information Used
1	Ground subsidence analysis	Z-axis coordinates
2	South wall deformation analysis of the central vault (CA)	verticality
3	Horizontal displacement analysis of the south wall of the CA	Y-axis coordinates
4	Moisture distribution analysis at the top of the north vault	Temperature value
5	Expression of results using digital measurement technology	Temperature value, verticality, intensity value, and RGB

## Data Availability

Requests for access to the data presented in the study should be made to corresponding author.
